# Central nervous system TB: a retrospective review of management

**DOI:** 10.5588/ijtldopen.25.0371

**Published:** 2025-10-10

**Authors:** V. Grey, G. Eather

**Affiliations:** ^1^ Metro South Clinical Tuberculosis Service, Princess Alexandra Hospital, Brisbane, Australia;; ^2^ The University of Queensland, School of Clinical Medicine, Australia;; ^3^ Australasian Clinical Tuberculosis Network Steering Committee.

**Keywords:** tuberculosis, CNS-TB, Queensland, Australia, high dose rifampicin

Dear Editor,

Central nervous system tuberculosis (CNS-TB) is a life-threatening complication of *Mycobacterium tuberculosis* (MTB) infection.^1^ Current research presents conflicting evidence about the optimal anti-TB medication and dosing for CNS-TB. In this study, we review the management and prescribing patterns of adult CNS-TB cases, with particular interest in use of high dose rifampicin (HDR).

This retrospective review identified all cases of CNS-TB from 1^st^ July 2002 to 31^st^ December 2022 in Queensland, Australia, using the Queensland Notifiable Conditions System. The search was limited to persons over the age of 14 as this is the cutoff value for adults in our centre. Confirmed diagnosis required either culturing MTB or detecting it through validated nucleic acid amplification testing in cerebrospinal fluid (CSF) or brain tissue. Presumed cases had compatible radiology and clinical presentation with either culture negative samples or growth of MTB at a non-CNS site. HDR was defined as doses over 600mg for adults over 50kg or over 450mg for adults under 50kg, as per Queensland’s statewide guidelines.^2^

Over the course of the study period, 65 adult cases of CNS-TB were notified in Queensland. 37 (57%) were male with a mean age of 37 (range 16–80) years. 7 (11%) died of CNS-TB. 45 (69%) completed treatment, 9 (14%) were transferred out of Australia and 3 (5%) were still on treatment at the time of data collection. 1 patient was lost to follow up. A total of 26 (40%) had a confirmed microbiological diagnosis. 17 patients cultured MTB on CSF and 2 patients on brain tissue. Nucleic acid amplification testing on culture negative samples detected MTB in an additional 6 patients on CSF and 1 patient on brain tissue. Including antibiotic susceptibility data from both CNS and non-CNS sites, 42 (89%) of these had fully susceptible disease. 2 (4%) of patients had multidrug resistant tuberculosis (MDR-TB), 2 (4%) had isoniazid monoresistance and 1 (2%) had rifampicin monoresistance.

11 patients (17%) received HDR initially. Excluding patients who were transferred out of Australia, 6 (12%) patients died who received standard dosing compared to 1 (14%) who received HDR. No adverse drug reactions occurred in patients who received HDR that could be directly attributed to using the higher than standard dose. The Table provides a comprehensive overview of the 11 patients who received HDR. Overall HDR was well tolerated. Initial concerns for drug induced hepatotoxicity in 3 patients resulted in a dose reduction to 600mg for 2 patients and a 1-week cessation in the third. In all these cases, liver function derangement was subsequently attributed to other causes. One patient developed a rash, which was later attributed to ethambutol and did not reoccur when HDR was restarted. HDR duration varied significantly (1 week to 18 months) based on the treating physician’s preference: 3 opted to only use initially (for 1, 2 and 8 weeks in each case) before dropping to standard dosing, with the rest choosing to continue for the entire treatment duration. Overall, 6 patients in the HDR cohort completed treatment, 4 were transferred out of Australia and 1 of them died. 10 patients received a fluoroquinolone empirically, exclusively moxifloxacin at doses between 400mg – 800mg daily. 7 completed treatment and 3 were transferred out of Australia whilst still on treatment. 4 of these 10 also received HDR.

**Table.** A comprehensive overview of the 11 patients who received high-dose rifampicin (HDR), detailing their demographic information, clinical characteristics, treatment parameters, and outcomes.. Y= Yes; N = No.

Patient	Country of birth	Age at diagnosis	HIV status	ICU admission	Mortality	Drug susceptibility	Dose of rifampicin	Drug interruption required whilst on HDR?	Duration of HDR	Adverse drug reaction?	Treatment outcome
1	Thailand	35	Negative	Y	N	Culture negative	900mg ∼13mg/kg/day	N	11 months	Possible hepatotoxicity.	Completed treatment
2	Papua New Guinea	53	Positive	Y	N	Fully sensitive	900mg ∼12mg/kg/day	Y – 1 week then successfully re introduced	At least 8 months	Possible hepatotoxicity	Transferred out of Australia
3	Australia	36	Negative	N	N	Fully sensitive	900mg ∼8mg/kg/day	Y – due to compliance issues	At least 10 months	N	Completed treatment
4	Congo	32	Negative	Y	Y	Fully sensitive	900mg ∼16mg/kg/day	N	1 week	Possible hepatotoxicity	Died of TB
5	India	33	Negative	Y	N	Fully sensitive	900mg ∼18mg/kg/day	N	1 week	N	Transferred out of Australia
6	Nepal	56	Negative	N	N	Culture negative	900mg ∼11mg/kg/day	N	2 months	N	Completed treatment
7	Papua New Guinea	23	Negative	Y	N	Culture negative	900mg ∼16mg/kg/day	N	At least 12 days	N	Transferred out of Australia
8	Papua New Guinea	22	Negative	Y	N	Fully sensitive	750mg ∼14mg/kg/day	N	At least 6 weeks	N	Transferred out of Australia
9	Bangladesh	40	Negative	N	N	Culture negative	900mg ∼19mg/kg/day	N	18 months	N	Completed treatment
10	China	49	Negative	N	N	Culture negative	900mg ∼14mg/kg/day	Y	14 months	Rash	Completed treatment
11	Papua New Guinea	44	Negative	N	N	Fully sensitive	900mg ∼11mg/kg/day	N	2 weeks	N	Completed treatment

CNS-TB management showed substantial therapeutic uncertainties and prescribing variability, particularly regarding HDR and fluoroquinolone use. Only 17% of patients received HDR, with doses ranging from 750–900mg. This reflects the ongoing uncertainty about optimal dosing strategies. Despite concerns about potential toxicity, our data suggested HDR was relatively well-tolerated. Duration of HDR also varied greatly, which mirrors the lack of consensus around this internationally. 10 patients received a fluoroquinolone at the start of therapy with moxifloxacin chosen at variable doses. This again represents a small proportion of total case number and reflecting the lack of clarity regarding its clinical utility. Established in 1971, the standard 600mg rifampicin dose, has been increasingly questioned due to its limited CSF penetration.^3^ In 2016, a randomised control trial (RCT) of 817 adults in Vietnam found no significant mortality or neurological disability benefit when using rifampicin doses of 15mg/kg with levofloxacin compared to standard rifampicin dosing.^4^ Higher frequency of seizures was seen in the intensified group and more significant bilirubin elevations. The authors investigated the impact of using HDR and levofloxacin together, so it is not possible to draw conclusions on safety of HDR from this study. Ruslami et al., conducted an open label phase 2 trial where 60 participants were randomised to receive either standard oral dosing or higher intravenous dosing of rifampicin with either standard dose, HDR or no moxifloxacin with standard therapy for the first two weeks of treatment.^5^ 6-month mortality was significantly lower in patients who received intravenous HDR (10 [35%] versus 20 [65%]) and no increased toxicity was seen. This study may have been influenced by some physicians from Queensland to only use HDR in the initial intensive period rather than for the whole treatment course. Empirical use of a fluoroquinolone in CNS-TB has been suggested to be of benefit given high CSF concentrations reached. A systematic review and meta-analysis of 5 trials with 1115 patients with CNS-TB found no additional mortality benefit when a fluoroquinolone was used.^6^ There was also a high incidence of vision loss and seizures when used. There is no known clinical trial data to date that indicate fluoroquinolones improve survival in fully sensitive CNS-TB.^7^ These studies demonstrate why practice variability exists for HDR and fluoroquinolones use, and emphasise the importance of evaluating both safety and effectiveness in larger populations. Significant variability exists in what constitutes HDR and guideline consensus is lacking. Further phase III randomised controlled trials are underway to help guide therapeutic choices in CNS-TB and hopefully will address the unanswered questions.^7,8^

This study had several limitations. First, it was retrospective in nature. Second, despite the 20.5 year study period, only a relatively small number of CNS-TB cases were notified and an even smaller number received HDR. This makes clinical practice recommendations difficult. However, the analysis does highlight the data gap and the importance for further review.

Although the study suggests HDR may be safe, the small sample size prevents definitive conclusions. International studies are conflicting regarding a potential mortality benefit from HDR. Prescribing patterns were highly variable across the state; given CNS-TB has a significant risk of mortality, there is urgent need for further research to help build evidence-based guidelines to ensure a standardized approach to care.
